# Impacts of Entrepreneurial Openness and Creativity on Company Growth

**DOI:** 10.3389/fpsyg.2022.860382

**Published:** 2022-06-30

**Authors:** Žiga Peljko, Jasna Auer Antončič

**Affiliations:** ^1^KD Group d.d., Ljubljana, Slovenia; ^2^Faculty of Management, University of Primorska, Koper, Slovenia

**Keywords:** entrepreneurial openness, creativity, entrepreneur, personality, growth, entrepreneurship, SMEs

## Abstract

Entrepreneurs as individuals are the main drivers of entrepreneurship and possess distinct personality characteristics. The study focused on entrepreneurial openness and creativity on the entrepreneurial level relative to business growth. Hypotheses were developed and empirically tested in structural equation models using survey data obtained from SMEs’ entrepreneurs in three countries. This study adds to what is known about entrepreneurship and small business management in terms of normative research on firm growth by empirically examining the relationships between the entrepreneurial openness, creative personality, and creativity of the entrepreneur and growth of the company. Moreover, the study develops refined internationally comparable measures of entrepreneurial openness, entrepreneur creativity, and a creative personality. An entrepreneur’s openness and creative personality may be essential for their creativity. The entrepreneur’s creativity may be a vital element of company growth in some countries.

## Introduction

Entrepreneurs as individuals are the main drivers of entrepreneurship. The personality characteristics of entrepreneurs mean they can make a difference in how their companies perform (Antončič et al., 2018). Despite the notion that variations in performance among companies can be explained by distinct differences among those individuals who lead companies, there is not much research in this area (Mollick, 2012), except for studies that consider individual differences while studying firm performance, like studies investigating managers (Lieberson and O'Connor, 1972; Bertrand and Schoar, 2003; Crossland and Hambrick, 2011; Mollick, 2012) or entrepreneurs (Gimeno et al., 1997; Johnson, 2007; Antončič et al., 2018).

Much research in the entrepreneurship field relies on the assumption that entrepreneurs have distinct personality characteristics that can be identified (Cooper and Dunkelberg, 1987). Personality traits constitutes a broad field attracting intensive research, where it is shown they are able to influence organizations. For example, Ruzzier et al. (2007) state that entrepreneurs draw on their human capital (knowledge, skills, values) to advance the interests of their organization.

Researchers have considered a range of determinants that affect entrepreneurial behavior, such as creativity (Shalley, 1991; Ward, 2004; DiLiello and Houghton, 2006) and the Big Five personality factors (extraversion, agreeableness, conscientiousness, neuroticism, openness to experience; Ciavarella et al., 2004; Zhao et al., 2010; Antončič et al., 2015). It makes sense to examine how entrepreneurial openness affects creativity on the level of the individual—the entrepreneur. Openness to experience is a typical element of entrepreneurship (Singh and DeNoble, 2003). Individuals possessing a high level of openness to experience are tolerant of ambiguity and able to create distant and unusual associations (McCrae, 2007), which may help in discovering entrepreneurial ideas. We study the construct of entrepreneurial openness, which is a specialized measure of openness in this area of study and thus suitable for consideration. The construct of entrepreneurial openness was developed by Slavec et al. (2017) and helps to understand the impact an entrepreneur’s personality has on the performance of their SMEs. In this article, we are interested in whether entrepreneurial openness influences creativity on the level of entrepreneurs such that they can successfully generate useful ideas and solutions and thereby influence the growth of their business. This study intends to fill a gap in the research on creativity and firm-level performance given that only a few studies have assessed this relationship (for example, Von Nordenflycht, 2007; Weinzimmer et al., 2011; Khedhaouria et al., 2015), but without taking account of the entrepreneur’s creativity, creative personality and entrepreneurial openness together in a model.

Edwards-Schachter et al. (2015) note that while creativity, innovation, and entrepreneurship are recognized as key ingredients for fostering an entrepreneurial culture, their relationship to a skills-based approach remains insufficiently understood. Creativity is the premise of individual geniuses (Perry-Smith and Mannucci, 2015), while creativity and entrepreneurship are closely connected (Tiwari and Verma, 2020). Creativity may be understood as the creation of new ideas and innovations as well as the commercialization of new ideas (Basadur, 2004), which promotes an entrepreneurial culture (Edwards-Schachter et al., 2015), meaning it is reasonable to explore whether an individual entrepreneur’s creativity affects the growth of their company. In summary, this study concentrated on entrepreneurial openness and creativity on the entrepreneurial level relative to business growth.

## Theory and Hypotheses

Growth of the company may be regarded as a key concept in entrepreneurship because entrepreneurship can be growth and growth can be entrepreneurship (Davidsson et al., 2006). The growth of a company has two main connotations: (1) an increase in the amount (of its output, exports and/or sales) and (2) an increase in its size or an improvement in quality of its operations/products/services due a development process (Penrose, 1959, in Davidsson et al., 2010). Company growth typically indicates entrepreneurial success (Gupta et al., 2013) and is essential for economic development and the creation of wealth and employment, being best assessed in both absolute and relative terms (Davidsson and Wiklund, 2006). Growth of the company (including growth in employee numbers, sales and in market share) is often considered an important element of the company’s performance (Antončič and Hisrich, 2001). Sales growth may be an appropriate measure of growth in the company’s performance because it reflects stronger demand for the company’s products/services (Wiklund, 1999).

The right set of entrepreneurial characteristics can boost the results of entrepreneurial activities (for example, Zhao et al., 2010; Obschonka et al., 2013; Antončič et al., 2018). For instance, Ayala and Manzano (2014) note that three dimensions of entrepreneurial flexibility (courage, ingenuity, optimism) help predict entrepreneurial success, which might prove to be important for this study because flexibility is associated with creativity (Tasan-Kok, 2008). From the point of view of psychology, the constructs selected on the entrepreneurial level to be used in models of growth of the company are well connected and upgraded, one on top of the other.

One may conclude that entrepreneurs express quite considerable entrepreneurial openness that ensures their long-run success; entrepreneurs must be open to new things that can help them do business. Slavec et al. (2017) state that entrepreneurial openness consists of three categories: openness to learning, which directs entrepreneurs to learn about new ways of marketing and management approaches; openness to newness, which is crucial during the processes of innovation adoption; and openness to feedback, since entrepreneurs actively seek feedback to gain a competitive advantage, creatively solve problems, and reshape ideas to make them more relevant to market needs. Accordingly, we study how entrepreneurial openness, which we consider as an independent variable, affects the entrepreneur’s creativity.

Creativity is a very complex concept that can be defined, understood and applied in various ways. This means the term must be properly defined for this study’s purposes, relying on originality, usefulness, flexibility and mobility as the main criteria for creativity (Štemberger, 2013), whereas the focus is on creativity on the level of the individual entrepreneur. A creative-person approach is therefore used as it seeks to define general and specific abilities, motives and characteristics that describe an individual who makes creative products (Gough, 1979; Carroll, 1993; Eysenck, 1993; Batey and Furnham, 2006), while creativity will be treated as a personal characteristic.

Entrepreneurial creativity is considered in this study as both a dependent variable and partly as an independent one. Creativity is what distinguishes humans from other species, which probably explains this great interest in studying it on a general level (Ko and Butler, 2007). Creativity is directly related to entrepreneurship and entrepreneurs because when change is constant one must continually look for creative solutions to the current challenges. Entrepreneurs are creative in their work due to what is required or expected of them (Antonio et al., 2014). An increase in openness to experience can affect the relationship between quantity of ideas and creativity (Friis-Olivarius and Christensen, 2019). Shi et al. (2016) found positive relationships between openness to experience, intelligence and creative thinking in children in China. McCrae and Costa (1997) note that, on one hand, employees with a high level of openness to experience have access to different approaches and perspectives, and that entrepreneurial openness is a positive personality strength that includes characteristics like acquiring new skills, themes, and bodies of knowledge; discovering new and productive ways of doing things; and contemplating and studying things from all aspects (Slavec et al., 2017). We thus propose hypothesis 1, as follows:

*Hypothesis 1*: Entrepreneurial openness has a positive effect on the entrepreneur’s creativity.

The entrepreneur’s creativity may have another antecedent—a creative personality. A creative personality (Kaufman and Baer, 2004) entails general creativity on the level of the individual, whereas the creativity on the level of an entrepreneur (Puhakka, 2005) is more specific to the domain of entrepreneurship. Kaufman et al. (2009) developed the hierarchical creativity construct and found that its most reflective domains were performance and artistic/visual, with its much less reflective domains being math/science and problem-solving. Key items were identified (Kaufman et al., 2009) for each creativity domain: the entrepreneur domain (the most reflective items: advertising and business); the performance domain (acting and film); the math/science domain (life sciences and chemistry); the artistic/visual domain (painting and crafts); the problem-solving domain (mechanical and logic); the interpersonal domain (personal problems and interacting with one’s family); and the artistic/verbal one (writing fiction and writing nonfiction). Kaufman et al. (2009) state the question of whether creativity is general (a creative individual) or domain-specific (for example, a creative poet, a creative mathematician, a creative architect) is sometimes left unanswered or ignored. In this study, we include both aspects and hypothesize that people with a higher level of general creativity are more likely to develop a specific form of creativity in entrepreneurship:

*Hypothesis 2*: A creative personality has a positive effect on the entrepreneur’s creativity.

According to one definition, creativity is the imaginative recombination of elements from the past into new configurations needed in the present (Torrance, 1988). Kampylis et al. (2009) define creativity as an activity (both mental and physical) that occurs in a particular time, spatial, social and cultural context and leads to original tangible/intangible outcomes that are useful, ethical and desirable, if not for others then at least for the creator. This knowledge constitutes a scientific challenge on the level of entrepreneurship and thus in this study we explore how the creativity of entrepreneurs affects the growth of their companies. Trstenjak (1981) argued that creativity is built on two starting points in the relationship between the individual and society: the individual in cooperation with society, and vice versa when society encourages the individual.

Innovation (or innovativeness) is a defining ingredient of entrepreneurship (for example, Schumpeter, 1934, 1942) and is crucial for firm performance (for example, Antončič et al., 2007; Antončič and Prodan, 2008). Innovativeness is not included in this study, yet it must be distinguished from creativity. For the purposes of separating the constructs of creativity and innovation on the entrepreneurial level, we note the rationale given by Gurteen (1998) who states that a more useful definition of creativity is the process of generating ideas, while innovation should be seen as a treatment, improvement and, more critically, the implementation of these ideas. Gurteen (1998) listed several differences: creativity refers to divergent thinking, innovation refers to convergent thinking; creativity refers to generating ideas, innovation puts ideas into action. Being creative means seeing the same things as everyone else, but thinking about something other than what everyone else does (Krueger and Brazeal, 1994).

The relationship between digital creativity and individual academic performance of adolescents can be positive and mediated by parenting styles (Pérez-Fuentes et al., 2019). Peljko et al. (2017) examined the relationship between the entrepreneur’s creative abilities and firm growth and obtained mixed results (a positive relationship in a combined sample from Slovenia and the United States of America, yet no relationship in Serbia). Wdowiak et al. (2012) suggest that entrepreneurial behavior can be improved by developing values of the individual like creativity, striving for a challenging life, or autonomy, through the early life of individuals in education and in the family, based on the finding of positive relationships between values held by the individual and entrepreneurial skills in Austria, Poland and Slovenia. Entrepreneurs constantly deal with questions/challenges for which they have no answer. Here, the essence of creativity lies in inventing new and better ways to do things and address certain risks since new ideas can ensure delivery of the planned positive results (Zhou and George, 2001). Creativity is important for organizations’ competitiveness and success (Çekmecelioğlu and Özbağ, 2016) as well as entrepreneurial success (Tiwari and Verma, 2020). We thus posit the following hypothesis:

*Hypothesis 3*: The creativity of the entrepreneur has a positive effect on the growth of their company.

**Figure 1:**
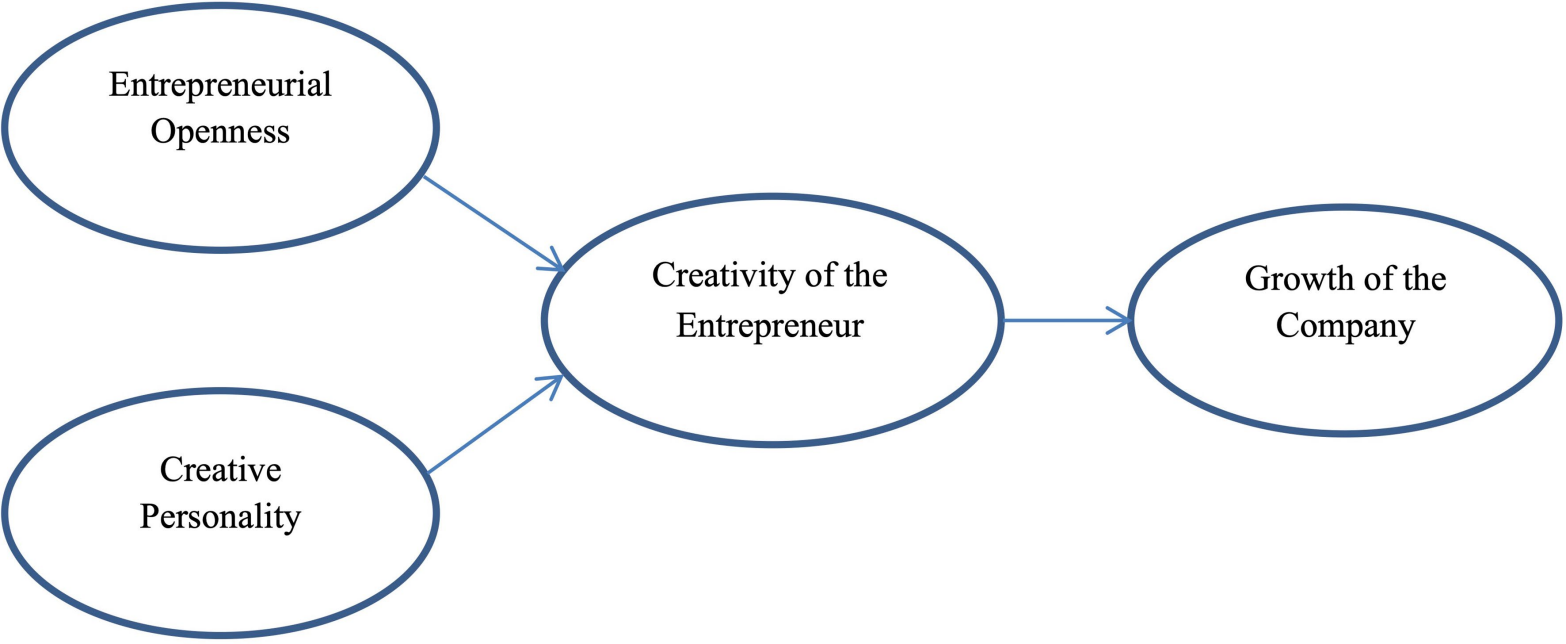
The model of entrepreneurial openness, creativity of the entrepreneur, and growth of the company.

The structural model discussed and verified in this research and reflected in hypotheses H1, H2 and H3 presented in the section above is shown in Figure 1.

## Research Methods

### Participants

Data for this study were obtained through an online survey questionnaire. The survey questionnaire was sent in each country (Slovenia, Serbia and Latvia) to a random sample of companies (SMEs) with up to 250 employees by e-mail with a request the questionnaire be filled out by entrepreneurs (owners and/or founders) online or returned by e-mail. Contact e-mails of SMEs were selected from available databases of all companies in each country. Then a probability sampling procedure yielded a smaller number of e-mails, to which the request to fill out the questionnaire was sent in each country. The sample yielded 851 usable responses from entrepreneurs of SMEs with up to 250 employees in three countries: Slovenia (*n* = 359), Serbia (*n* = 154) and Latvia (*n* = 338). Characteristics of the sample are presented in Table 1.

**Table 1:** Characteristics of the sample.

Characteristics of the sample (***n*** = 851)	Group (***n***)
Country	Slovenia (359)
	Serbia (154)
	Latvia (338)
Gender	Male (387)
	Female (464)
Age	Younger—over 20–50 years (507)
	Older—over 50 years (344)
Education	Up to undergraduate (599)
	Graduate degree (252)
Founder or co-founder	Yes (564)
	No (287)
Owner or co-owner	Yes (640)
	No (211)
Industry	Manufacturing (149)
	Services (702)
Firm age	0–10 years (329)
	11 or more years (522)
Size	0–10 employees (631)
	11–250 employees (220)
Stage in the life cycle	Start-up—growth (312)
	Maturity and later (539)

The surveyed SMEs were found to be sufficiently representative after comparing the size structure of companies with up to 250 employees in each country between the total population and the sample. The sample firms were generally small (up to 50 employees: Slovenia 96%, Serbia 97%, Latvia 98%; up to EUR 4 million in annual sales: Slovenia 92%, Serbia 97%, Latvia 96%) and medium-aged (operating in business between 11 and 50 years in Slovenia and Serbia and between 6 and 20 years in Latvia) from various industries (with services prevailing). The sample entrepreneurs were well represented in terms of gender and age (a slight majority of females in Slovenia 63% and Latvia 51% and males 58% in Serbia; the majority over 40 years old: Slovenia 75%, Serbia 60%, Latvia 73%, younger ones also well represented).

### Instrument

The survey included four measures (measurement items in Appendices in Appendix 1–4): (1) entrepreneurial openness (Slavec et al., 2017; 11 questions); (2) creative personality (Kaufman and Baer, 2004; 10 questions), which covers general creativity on the level of the individual; (3) creativity on the level of the entrepreneur (Puhakka, 2005; five questions); and (4) growth of the company (Antončič and Hisrich, 2001; Antončič and Antončič, 2011; three items: growth in the number of employees, sales and market share). Control variables were also assessed: industry, company life cycle, gender, age, education, and questions about (co-)ownership and a (co-)founding role in the company.

### Procedure

The data from Slovenia were used to develop the model, while the data from Serbia and Latvia were used to validate the models developed on the first sample. The constructs were analyzed for internal consistency and validity (Cronbach’s alpha reliability analysis, exploratory and confirmatory factor analysis). SPSS and EQS were used to assess the constructs. The models and hypotheses were tested with structural equation modeling (EQS). EQS was selected because of its benefits (Bentler, 1995): structural modeling in EQS is made simple, consistent, technically advanced, and accurate. Control variables (e.g., industry: production and services; life cycle: early and late stages; gender: female and male) were used while assessing the model differences on the sub-samples.

## Empirical Results

### Factor Analysis and Reliability Results

The entrepreneurial openness construct was first tested using exploratory factor analysis (method: ML, rotation: Oblimin) on the three samples (Slovenia, Serbia, Latvia), with the results being presented in Table 2. Four items were retained for the analysis based on the size of the communalities and their factor loadings. The appropriateness of factor analysis was ascertained by examining the correlation matrix, where Bartlett’s test of sphericity was used for this purpose. Bartlett’s test examines the presence of correlations among the principal variables. In all three countries under study, Bartlett’s test was significant (*p* < 0.001), showing that the correlation matrix includes significant correlations. The KMO measure (Kaiser–Meyer–Olkin measure of sampling adequacy) also showed acceptable sampling adequacy results (0.77 in Slovenia, 0.72 in Serbia, 0.83 in Latvia). The Cronbach alpha reliability test results were very good (Slovenia 0.77, Serbia 0.69, Latvia 0.87).

**Table 2:** The entrepreneurial openness construct—factor analysis and reliability results.

Factor analysis		Sample				
**Exploratory (method: ML, rotation: Oblimin)**		**Slovenia (***n*** = 359)**		**Serbia (***n*** = 154)**	**Latvia (***n*** = 338)**	
KMO		0.77		0.72	0.83	
Bartlett test	Chi square	346.27		101.02	660.90	
	df	6		6	6	
	*p*	0.000		0.000	0.000	
Total variance explained		45.49%		37.43%	63.43%	
Reliability	Cronbach alpha	0.77		0.69	0.87	
**Items**	**Communalities after extraction**			**Factor loadings**		
**Entrepreneurial openness**	**Slovenia**	**Serbia**	**Latvia**	**Slovenia**	**Serbia**	**Latvia**
New marketing approaches	0.5	0.3	0.6	0.70	0.55	0.75
Ideas for new products/services	0.5	0.3	0.6	0.68	0.57	0.78
Examine changes	0.4	0.3	0.6	0.59	0.56	0.80
An open mind	0.5	0.5	0.7	0.72	0.74	0.81
**Confirmatory (method: ERLS)**		**Slovenia (***n*** = 359)**		**Serbia (***n*** = 154)**	**Latvia (***n*** = 338)**	
Model	Chi square	3.66		1.86	26.30	
	df	3		3	3	
	*p*	0.300		0.603	0.000	
Goodness-of-fit	NFI	0.99		0.98	0.97	
	TLI (NNFI)	0.99		1.00	0.94	
	IFI	0.99		1.00	0.97	
	RMSEA	0.03		0.00	0.15	
	CFI	1.00		1.00	0.97	
Composite reliability	Cronbach alpha	0.77		0.69	0.87	
	RHO	0.76		0.70	0.84	
Discriminant validity	Average variance extracted (AVE)	0.44		0.38	0.56	

Items (frequency of occurrence: 1 = never, 2 = very rarely, 3 = rarely, 4 = occasionally, 5 = often, 6 = very often, and 7 = always): I learn new marketing approaches. I look for ideas for new products or services. I carefully examine all changes proposed to me by others (for example, I search for additional information on how to introduce changes, etc.). In terms of business matters, I have an open mind (thinking outside of the box and evaluating all options).

Second, the entrepreneurial openness construct was tested using confirmatory factor analysis (method: ERLS) on the three samples (results in Table 2). The confirmatory factor analysis confirmed the results of the exploratory factor analysis. All items had high, positive and significant coefficients. The construct showed good internal consistency (Cronbach alpha reliability: Slovenia 0.77, Serbia 0.69, Latvia 0.87; RHO: Slovenia 0.76, Serbia 0.70, Latvia 0.84). The construct also showed good convergence (model goodness-of-fit indices: NFI: Slovenia 0.99, Serbia 0.98, Latvia 0.97; RMSEA: Slovenia 0.03, Serbia 0.00, Latvia 0.15; CFI: Slovenia 1.00, Serbia 1.00, Latvia 0.97). The construct showed good discriminant validity in Latvia [average variance extracted (AVE) over 0.50 in all three countries] and marginally acceptable in Slovenia and Serbia (AVE around 0.4 with composite reliability over 0.6, Lam, 2012).

The creativity of the entrepreneur construct was first tested using exploratory factor analysis (method: ML, rotation: Oblimin) on the three samples (Slovenia, Serbia, Latvia). The results are shown in Table 3. Four items were retained for the analysis based on the size of the communalities and their factor loadings. In all three countries under examination, Bartlett’s test was significant (*p* < 0.001), revealing that the correlation matrix includes significant correlations. The KMO measure (Kaiser–Meyer–Olkin measure of sampling adequacy) also showed acceptable sampling adequacy results (0.81 in Slovenia, 0.77 in Serbia, 0.75 in Latvia). The Cronbach alpha reliability test results were very good (Slovenia 0.85, Serbia 0.81, Latvia 0.80).

**Table 3:** The creativity of the entrepreneur construct—factor analysis results.

Factor analysis		Sample				
**Exploratory (method: ML, rotation: Oblimin)**		**Slovenia (***n*** = 359)**		**Serbia (***n*** = 154)**	**Latvia (***n*** = 338)**	
KMO		0.81		0.77	0.75	
Bartlett test	Chi square	634.72		228.18	540.87	
	df	6		6	6	
	*p*	0.000		0.000	0.000	
Total variance explained		60.07%		55.15%	55.21%	
Reliability	Cronbach alpha	0.85		0.81	0.80	
**Items**	**Communalities after extraction**			**Factor loadings**		
**Creativity of the entrepreneur**	**Slovenia**	**Serbia**	**Latvia**	**Slovenia**	**Serbia**	**Latvia**
Modifying	0.2	0.4	0.4	0.43	0.63	0.66
New solutions	0.4	0.5	0.2	0.64	0.72	0.37
Problems’ solutions	0.6	0.7	1.0	0.80	0.81	1.00
Plenty of ideas	0.7	0.6	0.5	0.81	0.80	0.73
**Confirmatory (method: ERLS)**		**Slovenia (***n*** = 359)**		**Serbia (***n*** = 154)**	**Latvia (***n*** = 338)**	
Model	Chi square	0.69		5.72	8.50	
	df	3		3	3	
	*p*	0.875		0.126	0.037	
Goodness-of-fit	NFI	1.00		0.97	0.98	
	TLI (NNFI)	1.00		0.97	0.98	
	IFI	1.00		0.99	0.99	
	RMSEA	0.00		0.08	0.07	
	CFI	1.00		0.99	0.99	
Composite reliability	Cronbach alpha	0.85		0.81	0.80	
	RHO	0.86		0.80	0.80	
Discriminant validity	Average variance extracted (AVE)	0.61		0.54	0.53	

Items (agreement with the statement: 1 = strongly disagree, 2 = moderately disagree, 3 = slightly disagree, 4 = neither agree nor disagree, 5 = slightly agree, 6 = moderately agree, and 7 = strongly agree): I am good at modifying normal ways of doing things. New solutions come to my mind even if they are not especially needed. I come up with exceptional and surprising solutions to problems. I have plenty of ideas.

Second, the creativity of the entrepreneur construct was tested using confirmatory factor analysis (method: ERLS) on the three samples (results in Table 3). The confirmatory factor analysis corroborated the results of the exploratory factor analysis. All items had high, positive and significant coefficients. The construct showed good internal consistency (Cronbach alpha reliability: Slovenia 0.85, Serbia 0.81, Latvia 0.80; RHO: Slovenia 0.86, Serbia 0.80, Latvia 0.80). The construct also showed good convergence (model goodness-of-fit indices: NFI: Slovenia 1.00, Serbia 0.97, Latvia 0.98; RMSEA: Slovenia 0.00, Serbia 0.08, Latvia 0.07; CFI: Slovenia 1.00, Serbia 0.99, Latvia 0.99). The construct showed good discriminant validity (AVE over 0.50 in all three countries).

The creative personality construct was first tested using exploratory factor analysis (method: ML, rotation: Oblimin) on the three samples, with the results being presented in Table 4. Three items were retained for the analysis based on the size of the communalities and their factor loadings. In all three countries under study, Bartlett’s test was significant (*p* < 0.001), revealing that the correlation matrix includes significant correlations. The KMO measure also showed acceptable sampling adequacy results (Slovenia 0.60, Serbia 0.67, Latvia 0.64). The Cronbach alpha reliability test results were moderate (Slovenia 0.71, Serbia 0.78, Latvia 0.83).

**Table 4:** The creative personality construct—factor analysis results.

Factor analysis		Sample	
**Exploratory (method: ML, rotation: Oblimin)**		**Slovenia (***n*** = 359)**		**Serbia (***n*** = 154)**	**Latvia (***n*** = 338)**	
KMO		0.60		0.67	0.64	
Bartlett test	Chi square	257.96		138.00	446.86	
	df	3		3	3	
	*p*	0.000		0.000	0.000	
Total variance explained		50.99%		56.21%	65.59%	
Reliability	Cronbach alpha	0.71		0.78	0.83	
**Items**	**Communalities after extraction**			**Factor loadings**		
**Creative personality**	**Slovenia**	**Serbia**	**Latvia**	**Slovenia**	**Serbia**	**Latvia**
Do strange things	0.2	0.3	0.4	0.42	0.59	0.66
Enjoy fantasy	0.8	0.6	1.0	0.92	0.88	1.00
Love to daydream	0.5	0.6	0.5	0.71	0.75	0.73
**Confirmatory (method: ERLS)**		**Slovenia (***n*** = 359)**		**Serbia (***n*** = 154)**	**Latvia (***n*** = 338)**	
Model	Chi square	9.35		0.38	0.41	
	df	1		1	2	
	*p*	0.002		0.535	0.815	
Goodness-of-fit	NFI	0.96		1.00	1.00	
	TLI (NNFI)	0.94		1.00	0.20	
	IFI	0.97		1.00	0.60	
	RMSEA	0.15		0.00	0.00	
	CFI	0.96		1.00	1.00	
Composite reliability	Cronbach alpha	0.71		0.78	0.83	
	RHO	0.79		0.78	0.85	
Discriminant validity	Average variance extracted (AVE)	0.56		0.55	0.65	

Items (agreement with the statement: 1 = strongly disagree, 2 = moderately disagree, 3 = slightly disagree, 4 = neither agree nor disagree, 5 = slightly agree, 6 = moderately agree, and 7 = strongly agree): I do things that others find strange. I enjoy wild flights of fantasy. I love to daydream.

Second, the creative personality construct was tested using confirmatory factor analysis (method: ERLS) on the three samples (results in Table 4). The confirmatory factor analysis confirmed the results of the exploratory factor analysis. All items had high, positive and significant coefficients. The construct showed good internal consistency (Cronbach alpha reliability: Slovenia 0.71, Serbia 0.78, Latvia 0.83; RHO: Slovenia 0.79, Serbia 0.78, Latvia 0.85). The construct also showed good convergence (model goodness-of-fit indices: NFI: Slovenia 0.96, Serbia 1.00, Latvia 1.00; RMSEA: Slovenia 0.15, Serbia 0.00, Latvia 0.00; CFI: Slovenia 0.96, Serbia 1.00, Latvia 1.00). The construct showed good discriminant validity (AVE over 0.50 in all three countries).

The firm growth construct was initially tested using exploratory factor analysis (method: ML, rotation: Oblimin) on the three samples. The results are presented in Table 5. All three items were retained for the analysis based on the size of the communalities and their factor loadings. In all three countries under examination, Bartlett’s test was significant (*p* < 0.001). The KMO measure also showed acceptable sampling adequacy results (Slovenia 0.66, Serbia 0.64, Latvia 0.58). The Cronbach alpha reliability test results were good (Slovenia 0.70, Serbia 0.70, Latvia 0.67).

**Table 5:** Firm growth construct—factor analysis results.

Factor analysis		Sample	
**Exploratory (method: ML, rotation: Oblimin)**		**Slovenia (***n*** = 359)**		**Serbia (***n*** = 154)**	**Latvia (***n*** = 338)**	
KMO		0.66		0.64	0.58	
Bartlett test	Chi square	204.50		85.85	198.63	
	df	3		3	3	
	*p*	0.000		0.000	0.000	
Total variance explained		46.01%		46.40%	50.09%	
Reliability	Cronbach alpha	0.70		0.70	0.67	
**Items**	**Communalities after extraction**			**Factor loadings**		
**Growth**	**Slovenia**	**Serbia**	**Latvia**	**Slovenia**	**Serbia**	**Latvia**
No. of employees	0.3	0.3	0.2	0.59	0.56	0.41
Sales	0.6	0.7	1.0	0.79	0.85	1.00
Market share	0.4	0.4	0.3	0.64	0.59	0.58
**Confirmatory (method: ERLS)**		**Slovenia (***n*** = 359)**		**Serbia (***n*** = 154)**	**Latvia (***n*** = 338)**	
Model	Chi square	6.65		2.91	36.21	
	df	1		1	1	
	*p*	0.010		0.088	0.000	
Goodness-of-fit	NFI	0.96		0.97	0.68	
	TLI (NNFI)	0.90		0.94	0.03	
	IFI	0.97		0.98	0.68	
	RMSEA	0.13		0.11	0.32	
	CFI	0.97		0.98	0.68	
Composite reliability	Cronbach alpha	0.70		0.70	0.67	
	RHO	0.76		0.75	0.84	
Discriminant validity	Average variance extracted (AVE)	0.51		0.50	0.63	

Items: average annual growth in number of employees over the last 3 years (1 = less than 0%, 2 = 0%–4%, 3 = 5%–9%, 4 = 10%–19%, 5 = 20%–35%, 6 = more than 35%). Average annual growth in sales over the last 3 years (1 = less than 5%, 2 = 5%–9%, 3 = 10%–19%, 4 = 20%–34%, 5 = 35%–50%, 6 = more than 50%). Growth in market share over the last 3 years (the market share of your company is … 1 = decreasing, 2 = holding its own, 3 = increasing slightly, 4 = increasing moderately, and 5 = increasing significantly).

The firm growth construct was then tested using confirmatory factor analysis (method: ERLS) on the three samples (results in Table 5). The confirmatory factor analysis supported the results of the exploratory factor analysis. All items had high, positive and significant coefficients. The construct showed good internal consistency (Cronbach alpha reliability: Slovenia 0.70, Serbia 0.70, Latvia 0.67; RHO: Slovenia 0.76, Serbia 0.75, Latvia 0.84). The construct also showed good convergence in Slovenia and Serbia and somewhat less good convergence in Latvia (model goodness-of-fit indices: NFI: Slovenia 0.96, Serbia 0.97, Latvia 0.68; RMSEA: Slovenia 0.13, Serbia 0.11, Latvia 0.32; CFI: Slovenia 0.97, Serbia 0.98, Latvia 0.68). The construct showed good discriminant validity (AVE over 0.50 in all three countries).

### Non-response Bias and Common Method Bias Tests

Non-response bias was tested by applying the approach of Armstrong and Overton (1977), who stated that late respondents can be more like non-respondents. All model items’ means were compared for each country between these two groups (early respondents and late respondents), formed by using a median split based on the response time. Only for a few items in t-tests were significant mean differences found (the items “I enjoy wild flights of fantasy,” “I love to daydream,” and “growth in market share” in Slovenia, one item “new solutions come to my mind even if they are not especially needed” in Serbia, and no item in Latvia). These results indicate minimal response bias in this study.

Common method bias was tested using the approach of Harman (1976), for which Podsakoff and Organ (1986) suggested that common method bias can be assessed by applying the 50% threshold of total variance extracted in the one-factor test. All model items were included for each country in factor analysis with one fixed factor by using the principal components method of extraction. The total variance explained by a single factor was found to be below the 50% threshold in all three countries (33.9% in Slovenia, 30.0% in Serbia, 24.1% in Latvia), therefore the common method bias was not found to be present in this study.

### Structural Equation Modeling Results

The hypothesized relationships were tested in a model with structural equation modeling (method: ERLS) on both the overall data and the three samples (results shown in Tables 6, 7). The models were established to be appropriate both overall and in all three countries (model goodness-of-fit indices: NFI: overall 0.95, Slovenia 0.92, Serbia 0.90, Latvia 1.00; RMSEA: overall 0.06, Slovenia 0.07, Serbia 0.04, Latvia 0.03; CFI: overall 0.96, Slovenia 0.95, Serbia 0.98, Latvia 1.00; internal consistency: Cronbach alpha reliability: overall 0.79, Slovenia 0.83, Serbia 0.79, Latvia 0.74; RHO: overall 0.87, Slovenia 0.89, Serbia 0.86, Latvia 0.86).

**Table 6:** Structural equation modeling results (standardized coefficients and variance explained).

Sample (***n***)	EO-CE	CP-CE	CE-GR	R2CE	R2GR
Total (851)	0.39^***^	0.27^***^	0.13^**^	0.25	0.02
Slovenia (359)	0.64^***^	0.21^***^	0.23^***^	0.54	0.05
Serbia (154)	0.51^***^	0.34^***^	0.23^*^	0.47	0.05
Latvia (338)	0.14^*^	0.15^*^	-0.03	0.04	0.00

EO-CE: the entrepreneurial openness–creativity of the entrepreneur relationship coefficient. CP-CE: the creative personality–creativity of the entrepreneur relationship coefficient. CE-GR: the creativity of the entrepreneur–growth relationship coefficient. R2CE: variance explained (R-squared) of the creativity of the entrepreneur. R2GR: variance explained (R-squared) of the firm’s growth.

* *p* < 0.05 (two-sided);

** *p* < 0.01 (two-sided);

*** *p* < 0.001 (two-sided).

**Table 7:** Structural equation modeling results (goodness-of-fit and reliability).

Sample (***n***)	Chi	df	Sig.	NFI	RMSEA	CFI	RHO	Cronbach alpha
Total (851)	266.28	75	0.000	0.95	0.06	0.96	0.87	0.79
Slovenia (359)	219.45	75	0.000	0.92	0.07	0.95	0.89	0.83
Serbia (154)	96.59	75	0.047	0.90	0.04	0.98	0.86	0.79
Latvia (338)	105.48	77	0.017	1.00	0.03	1.00	0.86	0.74

Hypothesis 1 predicted a positive relationship between entrepreneurial openness and the creativity of the entrepreneur. Coefficients were found positive and significant overall and in all three countries (standardized coefficients: overall 0.39, Slovenia 0.64, Serbia 0.51, Latvia 0.14). These results act to support H1.

Hypothesis 2 predicted a positive relationship between a creative personality and the creativity of the entrepreneur. Coefficients were found positive and significant overall and in all three countries (standardized coefficients: overall 0.27, Slovenia 0.21, Serbia 0.34, Latvia 0.15). The results provide support for H2. Variance explained (R-squared) was found to be substantial overall (0.25), in Slovenia (0.54) and in Serbia (0.47), and lower in Latvia (0.04).

Hypothesis 3 predicted a positive relationship between the entrepreneur’s creativity and the growth of the firm. Coefficients were found to be positive and significant overall and in two of the three countries (standardized coefficients: overall 0.13, Slovenia 0.23, Serbia 0.23). Variance explained (R-squared) was found to be low overall (0.02), in Slovenia (0.05) and in Serbia (0.05), and non-existent in Latvia (0.00). The results mostly lend support for H3, except for Latvia.

The variability of the structural model results was tested by splitting the overall sample by control variables (results shown in Tables 8, 9). The entrepreneurial openness–creativity of the entrepreneur relationship coefficient was positive and significant on all control variables’ sub-samples, with one exception (positive and non-significant for the graduate education group), showing considerable support for H1. The creative personality–creativity of the entrepreneur relationship coefficient was positive and significant on all control variables’ sub-samples, showing a high level of support for H2. The creativity of the entrepreneur–growth relationship coefficient was positive on all sub-samples and significant in the majority of sub-samples, which means good support for H3. In addition to direct effects, some smaller indirect effects were detected in the model for the indirect effect of entrepreneurial openness and creative personality on growth through the creativity of the entrepreneur.

**Table 8:** Structural equation modeling results—controls (coefficients and variance explained).

Control group	(***n***)	EO-CE	CP-CE	CE-GR	R2CE	R2GR
Gender	Male (387)	0.22^*^	0.35^*^	0.14^*^	0.19	0.02
	Female (464)	0.50^*^	0.23^*^	0.11•	0.16	0.01
Age	Younger—over 20–50 years (507)	0.42^*^	0.25^*^	0.19^*^	0.28	0.01
	Older—over 50 years (344)	0.34^*^	0.31^*^	0.15^*^	0.24	0.02
Education	Up to undergraduate (599)	0.51^*^	0.26^*^	0.17^*^	0.38	0.03
	Graduate degree (252)	0.11	0.20^*^	0.03	0.06	0.00
Founder or co-founder	Yes (564)	0.45^*^	0.24^*^	0.19^*^	0.31	0.03
	No (287)	0.30^*^	0.28^*^	0.03	0.18	0.00
Owner or co-owner	Yes (640)	0.42^*^	0.26^*^	0.14^*^	0.28	0.02
	No (211)	0.33^*^	0.31^*^	0.09	0.22	0.01
Industry	Manufacturing (149)	0.32^*^	0.28^*^	0.18•	0.23	0.03
	Services (702)	0.40^*^	0.28^*^	0.11^*^	0.26	0.01
Firm age	0–10 years (329)	0.40^*^	0.22^*^	0.10	0.22	0.01
	11 or more years (522)	0.37^*^	0.30^*^	0.13^*^	0.27	0.02
Size	0–10 employees (631)	0.33^*^	0.26^*^	0.08	0.21	0.01
	11–250 employees (220)	0.50^*^	0.29^*^	0.17•	0.42	0.03
Stage in the life cycle	Start-up-growth (312)	0.47^*^	0.29^*^	0.20^*^	0.35	0.04
	Maturity and later (539)	0.33^*^	0.26^*^	0.06	0.20	0.00

EO-CE: the entrepreneurial openness–creativity of the entrepreneur relationship coefficient. CP-CE: the creative personality–creativity of the entrepreneur relationship coefficient. CE-GR: the creativity of the entrepreneur–growth relationship coefficient. R2CE: variance explained (R-squared) of the creativity of the entrepreneur. R2GR: the variance explained (R-squared) of the firm’s growth.

* *p* < 0.05 (two-sided);

• *p* < 0.10 (two-sided).

**Table 9:** Structural equation modeling results—controls (goodness-of-fit and reliability).

Control group	(***n***)	Chi	df	Sig.	NFI	RMSEA	CFI	RHO	Cronbach alpha
Gender	Male (387)	157.78	75	0.000	0.93	0.05	0.96	0.86	0.78
	Female (464)	190.72	75	0.000	0.94	0.06	0.96	0.87	0.80
Age	Younger—over 20–50 years (507)	194.47	75	0.000	0.94	0.06	0.96	0.86	0.79
	Older—over 50 years (344)	153.38	75	0.000	0.93	0.05	0.96	0.87	0.79
Education	Up to undergraduate (599)	224.74	75	0.000	0.95	0.06	0.96	0.88	0.81
	Graduate degree (252)	103.92	75	0.015	0.92	0.04	0.97	0.83	0.73
Founder or co-founder	Yes (564)	219.18	75	0.000	0.94	0.06	0.96	0.87	0.81
	No (287)	145.51	75	0.000	0.92	0.06	0.96	0.85	0.75
Owner or co-owner	Yes (640)	225.19	75	0.000	0.94	0.06	0.96	0.87	0.80
	No (211)	121.49	75	0.001	0.90	0.05	0.96	0.85	0.76
Industry	Manufacturing (149)	102.92	75	0.018	0.89	0.05	0.97	0.86	0.78
	Services (702)	230.08	75	0.000	0.95	0.05	0.96	0.87	0.79
Firm age	0–10 years (329)	167.91	75	0.000	0.92	0.06	0.95	0.86	0.79
	11 or more years (522)	172.61	75	0.000	0.94	0.05	0.97	0.87	0.79
Size	0–10 employees (631)	200.88	75	0.000	0.95	0.05	0.97	0.87	0.78
	11–250 employees (220)	137.67	75	0.000	0.90	0.06	0.95	0.86	0.80
Stage in the life cycle	Start-up-growth (312)	156.35	75	0.000	0.93	0.06	0.96	0.87	0.81
	Maturity and later (539)	191.09	75	0.000	0.94	0.05	0.96	0.86	0.77

## Discussion, Contributions, and Implications

A positive relationship between the entrepreneur’s entrepreneurial openness and their creativity was found in all three countries under study. Based on their openness (e.g., to learn new marketing approaches, look for ideas for new products or services, carefully examine all changes proposed by others, think outside of the box, evaluate all options), individuals will tend to develop entrepreneurial creativity (e.g., to become good at modifying the normal ways of doing things, new solutions come to mind even if not especially needed, inventing exceptional and surprising solutions for problems, and having plenty of ideas). A positive relationship between the entrepreneur’s creative personality and their creativity was established. The creative personality (e.g., doing things that others find strange, enjoying wild flights of fantasy, loving to daydream) can influence entrepreneurial creativity. We may conclude that entrepreneurial openness and creative personality may be important antecedents of the entrepreneur’s creativity. A positive relationship between the creativity of the entrepreneur and firm growth was found in two of the three countries (Slovenia and Serbia, but not in Latvia). The entrepreneur’s creativity can be an influential driver of their firm’s growth (growth in the number of employees, sales growth, growth in market share) in some countries (in Slovenia and Serbia in our study) and not in others (Latvia in our study). This might reflect differences in culture among the three countries in this study because national culture and its individual elements can influence entrepreneurial growth intentions (Leković and Berber, 2019).

The contribution to science made by this study is the conceptually developed and empirically tested model of entrepreneurial openness, creativity, and growth. This study makes a theoretical contribution by showing that the entrepreneur’s openness and possession of a creative personality may be important for the entrepreneur’s creativity and that this very creativity may be important for the growth of their firm. These results were found on samples of entrepreneurs from three European countries (Slovenia, Serbia, Latvia), except for the entrepreneurial creativity–growth relationship in Latvia. The empirical results based on the model of entrepreneurial openness, creativity, and firm growth contribute to the normative research on firm growth (for instance, research on creativity and firm performance: Von Nordenflycht, 2007; Weinzimmer et al., 2011; Khedhaouria et al., 2015) by revealing the importance of entrepreneurial openness and a creative personality for predicting the entrepreneur’s creativity, and the importance of the entrepreneur’s creativity for their firm’s growth.

The findings from this study extend research on entrepreneurial personality (e.g., Gimeno et al., 1997; Johnson, 2007; Antončič et al., 2018) and research on creativity and success (e.g., Çekmecelioğlu and Özbağ, 2016; Peljko et al., 2017; Tiwari and Verma, 2020) by adding constructs of entrepreneurial openness and creative personality in the model. The study confirms previous findings on the relationship between entrepreneurial openness and creativity (e.g., Shi et al., 2016; Friis-Olivarius and Christensen, 2019). The study clarifies the structure of constructs of entrepreneurial openness (Slavec et al., 2017), creative personality (Kaufman and Baer, 2004) and entrepreneur’s creativity (Puhakka, 2005) by testing the constructs on data from three countries.

This study contributes to comparative international entrepreneurship research because it involves a multi-country study of entrepreneurial activity that includes the four levels classified by Terjesen et al. (2016): individual, firm, industry and country. These levels were embodied in the following:

1. The individual level: characteristics of entrepreneurs related to entrepreneurial openness, creativity of the entrepreneur, a creative personality, and the individual controls of gender, age, education, a (co-)ownership and a (co-)founding role in the company.
2. The firm level: growth of the company and the company controls of size, age and life cycle.
3. The industry level: a control variable industry (production and services).
4. The country level: three different countries (Slovenia, Serbia, and Latvia).

The study also makes an empirical contribution by refining or retesting measures of entrepreneurial openness (Slavec et al., 2017), the entrepreneur’s creativity (Puhakka, 2005), and a creative personality (Kaufman and Baer, 2004) in three countries and revealing the key internationally comparable (etic) items:

1. Entrepreneurial openness items: I learn new marketing approaches. I look for ideas for new products or services. I carefully examine all changes proposed to me by others (for example, I search for additional information on how to introduce changes, etc.). In terms of business matters, I have an open mind (thinking outside of the box and evaluating all options).
2. Creativity of the entrepreneur items: I am good at modifying normally used ways of doing things. New solutions come to my mind even if they are not especially needed. I come up with exceptional and surprising solutions to problems. I have plenty of ideas.
3. Creative personality items: I do things that others find strange. I enjoy wild flights of fantasy. I love to daydream.

This study holds implications for theory, research and practice. Theory can better focus on entrepreneurial openness and creativity on the level of the entrepreneur in the prediction of firm growth. On one hand, company growth can depend on creative entrepreneurs who can spot opportunities for growth. Creativity may be connected to entrepreneurship because creativity stimulates the recognizing of new opportunities (for example, Shane, 2003; Gielnik et al., 2012). On the other hand, growth might not be about creativity but more about imitating or copying others (for example, Schmitz, 1989; Segerstrom, 1991; Szulanski and Jensen, 2008). With this study we have added some evidence to help resolve this controversy (creativity vs. copying) by focusing on creativity and showing that growth of the company can depend on creative entrepreneurs in Slovenia and Serbia and on copying or other factors in Latvia.

Researchers can use the three cross-nationally comparable measures (openness of the entrepreneur, creative personality, and creativity of the entrepreneur) in their research. Practitioners and policymakers must take into account that the personality of the entrepreneur as concerns their openness and creativity might be important for the growth of their company (growth in employee numbers, sales, and market share), meaning that education and training for companies and students must focus more strongly on developing the openness and creativity of individuals in order to improve business results (growth). Education and training should concentrate on developing entrepreneurial openness (in terms of learning new marketing approaches, searching for ideas for new products/services, searching for information on how to introduce changes, and thinking outside of the box), entrepreneurial creativity (in terms of encouraging numerous ideas, modifying the normal ways of doing things, searching for new solutions even if they are not needed, and coming up with exceptional and surprising solutions to problems), and a creative personality (in terms of encouraging wild fantasizing, daydreaming, and doing things that others find strange).

## Limitations and Future Research Possibilities

This study is not without its limitations. The main limitations are: (1) the model which is developed is a partial model in that only some psychological constructs of the entrepreneur were considered; for example, the creativity of the entrepreneur could have other antecedents: self-efficacy, internal locus of control, achievement, and materialism (Nisula and Olander, 2020), whereas growth of the company could have other antecedents: firm strategy factors (for example, customer orientation, competitor orientation, relationship coordination) and industry characteristics (for example, industry growth; Leischnig et al., 2016). (2) The use of closed-ended questions and perceptual measures in the questionnaire. (3) The possible indications (NFI = 1; RMSEA = 0.000) of saturations of some of the structural models. (4) Data were collected in the same time period and thus inferences about causality in the hypotheses were developed based on the literature and not directly verified. (5) Data from SMEs’ entrepreneurs were collected in three European countries and thus the results might not be fully relevant to all countries around the world.

For future research, we suggest: (1) the relationships between the constructs entrepreneurial openness, creative personality and creativity of the entrepreneur on the individual level and the growth construct (firm level) could be further examined in other countries, perhaps by adding some other variables and/or constructs. (2) The cross-country comparable measures employed in this study could be upgraded in future research. (3) Qualitative research techniques like in-depth interviews might improve knowledge about the content and functioning of the conversions of personal-level activities and aspirations (related to openness and creativity) to firm-level business results (growth).

## Conclusion

This study contributes to knowledge about entrepreneurship and small business management in terms of normative research on firm growth by empirically examining the relationships between the entrepreneurial openness, creative personality, and creativity of the entrepreneur and growth of their company. Further, the study developed refined cross-nationally comparable measures of entrepreneurial openness, creativity of the entrepreneur, and creative personality. The entrepreneur’ openness and creative personality may be essential for their creativity. The entrepreneur’s creativity may be vital for the growth of their company in some countries.

## Data Availability Statement

The datasets presented in this article will be made available by the authors upon request. Requests to access the datasets should be directed to ziga.peljko@kd-group.si; jasna.auer@fm-kp.si.

## Ethics Statement

Ethical review and approval was not required for the study on human participants in accordance with the local legislation and institutional requirements. Written informed consent for participation was not required for this study in accordance with the national legislation and the institutional requirements.

## Author Contributions

ŽP and JA developed the research project, carried out the data collection, and revised the manuscript. ŽP carried out the data analysis and wrote the first draft. All authors contributed to the article and approved the submitted version.

## Conflict of Interest

ŽP was employed by KD Group d.d.

The remaining author declares that the research was conducted in the absence of any commercial or financial relationships that could be construed as a potential conflict of interest.

## Publisher’s Note

All claims expressed in this article are solely those of the authors and do not necessarily represent those of their affiliated organizations, or those of the publisher, the editors and the reviewers. Any product that may be evaluated in this article, or claim that may be made by its manufacturer, is not guaranteed or endorsed by the publisher.

## Appendix

**Appendix 1:** Entrepreneurial openness items (Slavec et al., 2017).

	Frequency of occurrence: 1 = never, 2 = very rarely, 3 = rarely, 4 = occasionally, 5 = often, 6 = very often, 7 = always						
I follow successful entrepreneurs to learn something from them (I watch TV shows about successful entrepreneurs and/or attend their lectures and/or read articles about them).	1	2	3	4	5	6	7
I learn new marketing approaches.	1	2	3	4	5	6	7
I learn new approaches about managing the business.	1	2	3	4	5	6	7
I look for ideas for new products or services.	1	2	3	4	5	6	7
I look for new markets.	1	2	3	4	5	6	7
I look for new business partners.	1	2	3	4	5	6	7
I search for information about introducing my firm into new geographic markets.	1	2	3	4	5	6	7
I carefully examine all changes proposed to me by others (for example, I search for additional information on how to introduce changes, etc.).	1	2	3	4	5	6	7
I ask employees for their opinion on which improvements could be introduced.	1	2	3	4	5	6	7
In terms of business matters, I have an open mind (thinking outside of the box and evaluating all options).	1	2	3	4	5	6	7
In business, I search for creative solutions.	1	2	3	4	5	6	7

**Appendix 2:** Creative personality items (Kaufman and Baer, 2004).

	Accuracy of the statement: 1 = very inaccurate, 2 = inaccurate, 3 = moderately inaccurate, 4 = neither inaccurate nor accurate, 5 = moderately accurate, 6 = accurate, 7 = very accurate						
1. I do things that others find strange.	1	2	3	4	5	6	7
2. I like to get lost in thought.	1	2	3	4	5	6	7
3. I enjoy wild flights of fantasy.	1	2	3	4	5	6	7
4. I do things by the book.	1	2	3	4	5	6	7
5. I love to daydream.	1	2	3	4	5	6	7
6. I swim against the current.	1	2	3	4	5	6	7
7. I like to solve complex problems.	1	2	3	4	5	6	7
8. I am not interested in abstract ideas.	1	2	3	4	5	6	7
9. I love to read challenging material.	1	2	3	4	5	6	7
10. I have a vivid imagination.	1	2	3	4	5	6	7

**Appendix 3:** Creativity of the entrepreneur items (Puhakka, 2005).

	Agreement with the statement: 1 = strongly disagree, 2 = moderately disagree, 3 = slightly disagree, 4 = neither agree nor disagree, 5 = slightly agree, 6 = moderately agree, 7 = strongly agree						
1. I am good at modifying normally used ways of doing things.	1	2	3	4	5	6	7
2. I’m sensitive to seeing problems that others do not see.	1	2	3	4	5	6	7
3. New solutions come to my mind even if they are not especially needed.	1	2	3	4	5	6	7
4. I come up with exceptional and surprising solutions to problems.	1	2	3	4	5	6	7
5. I have plenty of ideas.	1	2	3	4	5	6	7

**Appendix 4:** Growth of the company items (Antončič and Hisrich, 2001; Antončič and Antončič, 2011).

Growth of your company		
1. Average annual growth in number of employees over the last 3 years:		
Less than 0%	0%–4%	5%–9%
10%–19%	20%–35%	More than 35%
2.Average annual growth in sales over the last 3 years:		
Less than 5%	5%–9%	10%–19%
20%–34%	35%–50%	More than 50%
3.Growth in market share over the last 3 years: the market share of your company is…		
Decreasing	Holding its own	Increasing slightly
Increasing moderately	Increasing significantly	
